# Redox-sensitive DNA binding by homodimeric *Methanosarcina acetivorans* MsvR is modulated by cysteine residues

**DOI:** 10.1186/1471-2180-13-163

**Published:** 2013-07-16

**Authors:** Catherine E Isom, Jessica L Turner, Daniel J Lessner, Elizabeth A Karr

**Affiliations:** 1 Department of Microbiology and Plant Biology, University of Oklahoma, 770 Van Vleet Oval, Norman, OK, 73019, USA; 2 Department of Biological Sciences, University of Arkansas-Fayetteville, Fayetteville, USA

**Keywords:** Methanogens, Transcription, Archaea, Regulation

## Abstract

**Background:**

Methanoarchaea are among the strictest known anaerobes, yet they can survive exposure to oxygen. The mechanisms by which they sense and respond to oxidizing conditions are unknown. MsvR is a transcription regulatory protein unique to the methanoarchaea. Initially identified and characterized in the methanogen *Methanothermobacter thermautotrophicus* (Mth), MthMsvR displays differential DNA binding under either oxidizing or reducing conditions. Since MthMsvR regulates a potential oxidative stress operon in *M. thermautotrophicus*, it was hypothesized that the MsvR family of proteins were redox-sensitive transcription regulators.

**Results:**

An MsvR homologue from the methanogen *Methanosarcina acetivorans*, MaMsvR, was overexpressed and purified. The two MsvR proteins bound the same DNA sequence motif found upstream of all known MsvR encoding genes, but unlike MthMsvR, MaMsvR did not bind the promoters of select genes involved in the oxidative stress response. Unlike MthMsvR that bound DNA under both non-reducing and reducing conditions, MaMsvR bound DNA only under reducing conditions. MaMsvR appeared as a dimer in gel filtration chromatography analysis and site-directed mutagenesis suggested that conserved cysteine residues within the V4R domain were involved in conformational rearrangements that impact DNA binding.

**Conclusions:**

Results presented herein suggest that homodimeric MaMsvR acts as a transcriptional repressor by binding Ma P_*msvR*_ under non-reducing conditions. Changing redox conditions promote conformational changes that abrogate binding to Ma P_*msvR*_ which likely leads to de-repression.

## Background

As the sole producers of biogenic methane, methanogenic *Archaea* (methanoarchaea) are a unique and poorly understood group of microorganisms. Methanoarchaea represent some of the most oxygen sensitive organisms identified to date [1], yet many methanogens can withstand oxygen exposure and resume growth once anaerobic conditions have been restored [2-4]. Thus, methanogens must have effective mechanisms for sensing and responding to redox changes in their local environment. Many methanogenic genomes encode homologues of proteins like superoxide dismutase, alkylhydroperoxide reductase, superoxide reducatase, and rubrerythrins that are known to combat oxidative stress in anaerobes [5-7]. Thus, methanogens potentially have several mechanisms for mitigating the damage caused by temporary oxidative stress. A better understanding of the oxidative stress response in methanogens is important for understanding their contributions to the planetary ecosystem.

At least one methanogenic protein, F_420_H_2_ oxidase, has been shown to reduce O_2_ to H_2_O [8]. In *Methanothermobacter thermautotrophicus,* F_420_H_2_ oxidase is the product of *fpaA* (MTH1350) whose promoter, P_*fpaA*_, is regulated by the methanogen-specific V4R domain regulator (MsvR). *M. thermautotrophicus* MsvR (MthMsvR) and its homologues are unique to a subset of methanogens, including the *Methanomicrobiales* and *Methanosarcinales*[9]. Besides controlling expression of *fpaA*, MthMsvR has also been shown to regulate its own expression at the transcriptional level *in vitro*. In its reduced state, MthMsvR represses transcription of *fpaA* and *msvR* by abrogating the binding of general transcription factors at the promoter, P_*fpaA*_ or P_*msvR*_, respectively [9].

Except for the use of a bacterial-like regulator, the basal transcriptional machinery of methanogens and all *Archaea* resembles that of eukaryotes. The multi-subunit RNA polymerase (RNAP) in *Archaea* resembles the eukaryotic RNAP II complex and is recruited to the promoter by homologues of the eukaryotic TATA binding protein (TBP) and TFIIB (TFB in *Archaea*). Archaeal transcription regulators can possess either activator or repressor functions and a few rare examples possess both functions [10]. The only clearly defined activation mechanism to date involves recruitment of TBP to the promoter [11], while archaeal repressors bound near the promoter have been shown to repress transcription in several ways, including abrogation of TBP/TFB or RNA polymerase binding to the promoter [10].

Consistent with its ability to differentially regulate transcription in response to changes in redox status, the domain architecture of MthMsvR and its homologues reveals both DNA binding and potential redox-sensitive functions. For example, MthMsvR has a classic bacterial helix-turn-helix DNA binding domain and a V4R domain. Although the V4R domain is present in many bacterial and archaeal proteins, the function of the V4R domain is not well understood and appears to have diverse functions from hydrocarbon binding to bacterio-chlorophyll synthesis [12]. There are three cysteine residues conserved within the V4R domain of MsvR family proteins. Earlier work with MthMsvR suggested differing DNA binding activity under oxidizing (or non-reducing) and reducing conditions [9]. Additionally, MthMsvR regulates expression of an operon encoding genes involved in oxidative stress response [5,8,9]. This suggests that the structure or function of the V4R domain in this family may be sensitive to cellular redox status.

Although homologues of MsvR are encoded in the majority of methanogen genomes, thus far, only MthMsvR has been characterized using *in vitro* approaches [9,13]. Currently, there are two genera of methanogens (*Methanococcus* and *Methanosarcina*) with genetically tractable species where *in vivo* approaches could be used to ascertain the role of MsvR [14,15]. The *in vitro* functional analysis of the *Methanosarcina acetivorans* MsvR (MaMsvR) homologue presented here opens the door for future *in vivo* analyses of the biological role of MsvR utilizing the genetic toolbox of *M. acetivorans*[16,17]. To determine whether the DNA-binding and redox-sensitive properties of MthMsvR are universal among MsvR homologues, the MsvR homologue (MA1458) from *M. acetivorans* (Ma) was purified and characterized.

## Results and discussion

### *M. acetivorans* C2A encodes an MsvR family protein, MaMsvR

**Figure 1:**
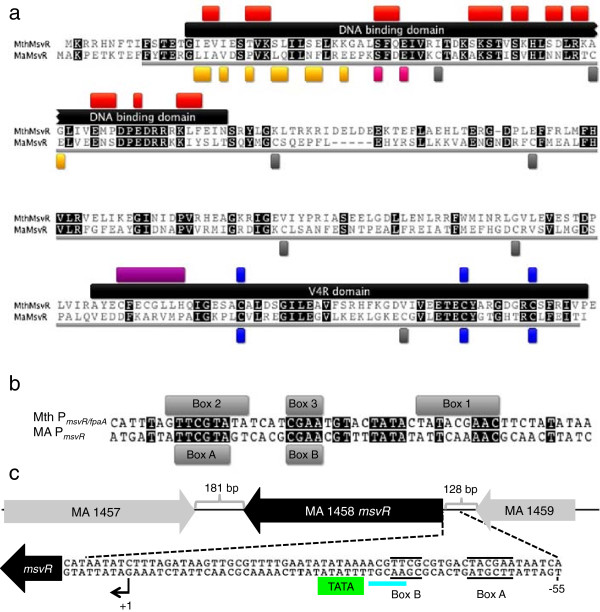
**Amino acid and intergenic alignments and genomic context.** (**a**) Amino acid alignment of *Methanothermobacter thermautotrophicus* (NP_276465.1) and *Methanosarcina acetivorans* C2A (NP_616392.1) MsvR proteins. Conserved residues are shaded black. The region of the alignment used to determine protein identity and similarity is underlined in gray. The DNA binding domain and V4R domain are represented by black boxes indicating the residues belonging to each domain. Red boxes indicate residues predicted to be involved directly in DNA binding whilst orange boxes indicate residues predicted to be involved in dimerization in both Ma and Mth MsvR. Residues within a predicted zinc binding domain in both Ma and Mth MsvR are represented by pink boxes [19]. Conserved cysteine residues are represented by blue boxes [pfam 02830, [19]]. Gray boxes identify additional cysteine residues in MaMsvR. A purple box indicates the CX_2_CX_3_H motif in MthMsvR. (**b**) Alignment of MsvR binding boxes in Ma P_*msvR*_ to those previously identified in Mth P_*msvR/fpaA*_[9]. Gray boxes indicate MsvR binding boxes 1, 2, and 3 on Mth P_*msvR/fpaA*_ and boxes A and B on Ma P_*msvR*_. Conserved nucleotides are shaded in black. (**c**) The genomic context of Ma *msvR* is illustrated (http://img.jgi.doe.gov, NCBI taxon ID 188937). Gray brackets identify intergenic regions and their corresponding lengths (181 bp and 128 bp). Dashed black outset lines identify the sequence of the region just upstream of Ma *msvR*. Green and turquoise boxes identify the *msvR* TATA box and B-recognition element, respectively. A bent arrow and the +1 designation indicate the mapped transcription start site of Ma *msvR*. The position of MsvR binding boxes A and B (solid black lines) in relationship to these two features is illustrated.

A BlastP [18] alignment indicated that at the amino acid level, MaMsvR is 33% identical and 48% similar to characterized MthMsvR (Figure 1a; >241 residues underlined in gray) [9]. The domain organization is also conserved between the two proteins, with an N-terminal DNA binding domain and a C-terminal V4R domain (Figure 1a). Within the DNA binding domain, 48% of the residues indicated by the conserved domain database (CDD) to be involved in DNA binding are conserved (Figure 1a, red boxes) and 45% of residues are conserved throughout the domain (Figure 1a, black box) [19]. Despite this disparity, all MsvR family proteins have a conserved DNA motif upstream of their MsvR encoding genes. In previous studies, this sequence was bound by MthMsvR [9]. Within the V4R domain, MthMsvR and MaMsvR are 36% identical. MthMsvR contains five cysteine residues, all within the V4R domain (Figure 1a, blue boxes, purple box) [9]. Two of the cysteines are found within a CX_2_CX_3_H motif characteristic of some metal-binding proteins involved in redox-sensitive transcription, such as the anti-sigma factor RsrA (Figure 1a, purple box) [20]. However, this motif is absent in MaMsvR, and in other MsvR homologues that do carry this motif, the histidine is replaced with a proline. The other three cysteine residues in the MthMsvR V4R domain are conserved in MaMsvR (Figure 1a, blue boxes). MaMsvR contains an additional seven cysteine residues, six of which lie outside the annotated V4R domain (Figure 1a, gray boxes). It is unlikely that the CX_2_CX_3_H motif in MthMsvR or the seven non-conserved cysteine residues (Figure 1a, gray boxes) in MaMsvR contribute to a shared regulatory mechanism in MsvR proteins. However, the three cysteine residues that are conserved in the V4R domains of MaMsvR and MthMsvR may be an important redox sensitive mechanism common to all MsvR family proteins.

### Genomic organization of Ma *msvR*

Mth *msvR* is transcribed divergently from an operon encoding three proteins involved in the oxidative stress response (http://img.jgi.doe.gov) (Figure 1c) [9]; thus, MthMsvR regulates expression from overlapping promoters. In contrast, Ma *msvR* (MA1458) is flanked by genes encoding an uncharacterized protein conserved in archaea (COG4044, MA1457) and a hypothetical protein with no conserved domains (MA1459) (Figure 1c) [19]. Therefore, MaMsvR only regulates its own promoter at this locus.

### Ma P_*msvR*_ and the location of MsvR binding boxes

MthMsvR has been shown to bind to at least three boxes on the shared intergenic region of Mth P_*msvR/fpaA*_[9]. The upstream region of known MsvR-encoding genes contains at least two of these binding boxes, suggesting that these boxes may serve as DNA recognition sequences for auto-regulation by the MsvR family proteins. The binding boxes for MthMsvR overlap the transcription start site in Mth P_*fpaA*_ and the BRE/TATA box in Mth P_*msvR*_*.* MthMsvR binding to box(es) two and three have been shown to prevent binding of TBP and TFB to Mth P_*msvR*_[9], suggesting that MthMsvR acts as a transcription repressor. Ma P_*msvR*_ contains two MsvR binding boxes, A and B, corresponding to Mth P_*msvR/fpaA*_ boxes 2 and 3, respectively (Figure 1b) [9]. In contrast to the seventy-three-nucleotide 5′ untranslated region (UTR) in the Mth *msvR* transcript [9], transcription start site mapping of the Ma *msvR* transcript indicates that transcription initiates at a G nucleotide eight nucleotides upstream of the ATG start codon (Figure 1c). The shorter 5′ UTR of Ma *msvR* is consistent with the results of transcription start site mapping in the closely related *Methanosarcina mazei* Gö1, where the *msvR* (MM2525) transcript was classified as leaderless for having a 5′ UTR of less than ten nucleotides [21]. A TATA box is centered 27 nucleotides upstream of the Ma *msvR* transcription start site and boxes A and B are located upstream of the TATA box (Figure 1c). MaMsvR binding to box B likely blocks the purine-rich BRE element just upstream of the Ma P_*msvR*_ TATA box, resulting in repression of transcription [9,10,22,23]. Despite some differences in the placement of the MsvR binding boxes, it is likely that MsvR proteins repress transcription of their own genes by blocking access to the promoter region.

### DNA binding behavior of MaMsvR varies under non-reducing and reducing conditions

**Figure 2:**
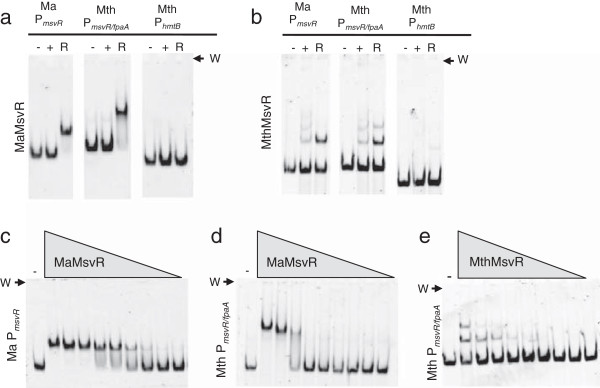
**EMSA of MsvR homologues on their respective promoters.** The gel wells are indicated (W). (**a** and **b**) EMSA to test binding of MaMsvR (**a**) and MthMsvR (**b**) to the MaMsvR promoter (Ma P_*msvR*_, 10 nM), the MthMsvR/*fpaA* intergenic promoter region (Mth P_*msvR/fpaA*_, 10 nM), and the Mth histone B promoter (Mth P_*hmtB*_, 10 nM). Each promoter has a control lane (-) that contains no protein, a binding reaction that contains either Ma or Mth MsvR (200 nM) in the absence of DTT (non-reduced, +), and a binding reaction that contains either Ma or Mth MsvR (200 nM) in the presence of 5 mM DTT (reduced, R). (**c**) EMSA assay (10 nM Ma P_*msvR*_ DNA) with decreasing concentrations of reduced MaMsvR (5 mM DTT) [monomer] 1 μM, 500 nM, 250 nM, 125 nM, 62.5 nM, 31.3 nM, 15.6 nM, 7.8 nM, and 3.9 nM. (**d**) EMSA assay (10 nM Mth P_*msvR/fpaA*_ DNA) with decreasing concentrations of reduced MaMsvR (5 mM DTT) [monomer] 1 μM, 500 nM, 250 nM, 125 nM, 62.5 nM, 31.3 nM, 15.6 nM, 7.8 nM, and 3.9 nM. (**e**) EMSA assay (10 nM Mth P_*msvR/fpaA*_ DNA) with decreasing concentrations of reduced MthMsvR (5 mM DTT) [monomer] 1 μM, 500 nM, 250 nM, 125 nM, 62.5 nM, 31.3 nM, 15.6 nM, 7.8 nM, and 3.9 nM.

Electrophoretic mobility shift assays (EMSAs) were used to compare the binding of MaMsvR to Ma P_*msvR*_ and Mth P_*msvR*/fpaA_ under non-reducing (+) and reducing (R) conditions (Figure 2a). Additionally, MthMsvR was tested for binding to Ma P_*msvR*_ and MthMsvR binding to Mth P_*msvR*/fpaA_ served as a control (Figure 2b). Both MaMsvR and MthMsvR bound to Ma P_*msvR*_ and Mth P_*msvR*/fpaA_. However, MaMsvR bound only under reducing conditions, while MthMsvR bound both promoters under non-reducing and reducing conditions (Figure 2a, b). This was consistent with previously published results showing that MthMsvR bound Mth P_*msvR/fpaA*_ under oxidizing and reducing conditions [9]. Neither protein showed notable binding to the well-described Mth histone control promoter (P_*hmtB*_), which demonstrated the specificity of MsvR binding (Figure 2a,b) [24,25].

The observed promoter binding behavior of MaMsvR is consistent with the hypothesis that MaMsvR acts as a transcription repressor of Ma P_*msvR*_ under reducing conditions. An oxidizing environment inhibits Ma P_*msvR*_ binding, likely leading to derepression. A mechanism for MthMsvR is less clear. Under reducing conditions, MthMsvR functions as a transcription repressor *in vitro*, yet MthMsvR binds the promoter under both reducing and non-reducing conditions. To reconcile this apparent discrepancy, it has been proposed that MthMsvR follows a mechanism reminiscent of the well-characterized redox regulator, OxyR, which binds DNA irrespective of redox status but has different effects on transcription under varying redox conditions [9,26]. These effects would likely be regulated by conformational changes in MthMsvR between the oxidized and reduced states. However, addressing this experimentally has been problematic because of both the limitations of the *M. thermautotrophicus in vitro* transcription system, which requires reducing conditions, and the complexity of the divergent promoter structure within Mth P_*msvR/fpaA*_.

### MaMsvR exhibits different DNA binding patterns than MthMsvR

MaMsvR appears to produce higher molecular weight complexes on Mth P_*msvR/fpaA*_ as movement of the DNA is further retarded in the gel compared to the shifted complex seen on Ma P_*msvR*_ (Figure 2a, c, and d). Consistent with previously published data, MthMsvR binding to Mth P_*msvR/fpaA*_ produced two distinct multiple shifted complexes, suggesting that varying stoichiometries of MthMsvR bound to Mth P_*msvR/fpaA*_ (Figure 2b) [9]. In contrast, only one shifted complex was seen with MaMsvR (Figure 2a, c, and d). To determine if MaMsvR was capable of producing complexes of varying stoichiometry, increasing concentrations of MaMsvR were incubated with Ma P_*msvR*_ (Figure 2c) or Mth P_*msvR/fpaA*_ (Figure 2d). Even at concentrations of one hundred-fold excess MaMsvR over DNA, only a single shifted complex was observed for either promoter. Conversely, at similar concentrations MthMsvR showed a binding pattern indicative of sequential addition of MthMsvR units, producing complexes of varying stoichiometries and thus varying molecular weights on Mth P_*msvR/fpaA*_ (Figure 2e) [27]. These results demonstrate differences in the stoichiometry of the protein:DNA complexes produced by MaMsvR and MthMsvR and suggests that the modes of oligomerization upon DNA binding may differ between the two proteins.

### MaMsvR binds an inverted repeat sequence conserved in all *msvR* promoters

**Figure 3:**
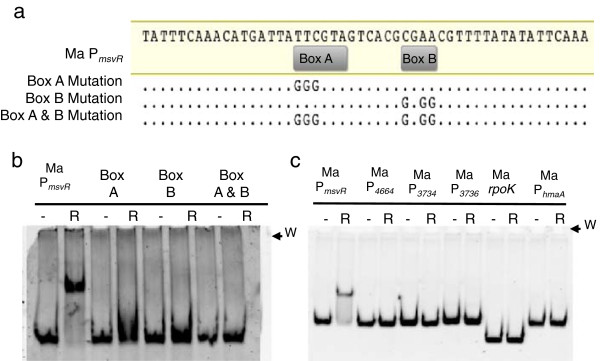
**MsvR binding and regulatory targets assessed by EMSA.** (**a**) Sequences of the 50 bp region of Ma P_*msvR*_ used to confirm MaMsvR binding to boxes A and B. Sequence changes within the binding boxes are shown. (**b**) EMSA assays with the template (50 nM) variations shown in (**a**) and 1 μM (20-fold excess over DNA) reduced MaMsvR (R, 5 mM DTT). A 50 bp region of Ma P_*msvR*_ was included as a binding control. The gel wells are indicated (W). (**c**) EMSA analysis with reduced MaMsvR (R, 5 mM DTT) and its own promoter (Ma P_*msvR*_, 10 nM), various intergenic regions of an oxidative stress response cluster (Ma P_*4664*_, P_*3734*_, P_*3736*_, 10 nM) as well as the control Ma histone A promoter (Ma P_*hmaA*_, 10 nM). A region of *rpoK* (10 nM) was tested for binding because an MsvR binding site (TTCGN_8_CGAA) is present in the coding region. The gel wells are indicated (W).

The two MsvR binding boxes in Ma P_*msvR*_, Boxes A and B, are found upstream of all known MsvR-encoding genes (Figure 1b,c; Figure 3a). Mth P_*msvR/fpaA*_ boxes 2 and 3, corresponding to Ma P_*msvR*_ boxes A and B represent a partial inverted repeat *TTCG*TAN_4_TA*CGAA*, whereas Mth P_*msvR/fpaA*_ Box 1 is a partial direct repeat of Box 3. The numbering of the boxes is based on order of discovery and not the order of MsvR binding. These binding boxes were previously identified by sequence alignments and their role in MthMsvR binding to Mth P_*msvR/fpaA*_ has been described [9]. MthMsvR complexes bound to all three boxes and DNaseI footprinting indicated involvement of upstream regions in conjunction with Box 1[9]. To determine if boxes A and B in Ma P_*msvR*_ were bound by MaMsvR, EMSAs were performed with fifty base-pair oligonucleotides spanning the binding boxes of Ma P_*msvR*_ (Figure 3). Mutations in either box A or box B eliminated MaMsvR binding, suggesting that this conserved sequence motif is involved in MsvR binding and auto-regulation (Figure 3b) [9]. Additionally, EMSA experiments with a single insertion or deletion between boxes A and B had no impact on MaMsvR binding suggesting that minor changes in spacing can be accommodated and that MaMsvR binding sites in the genome could be represented by the *TTCG*N_7-9_*CGAA* motif (see Additional file 1: Figure S1). There are over forty occurrences of such a motif upstream of structural genes in *M. acetivorans*. The structural genes are annotated to encode proteins involved in a variety of cellular functions including iron transport, divalent cation transport, efflux pumps, control of cell division, and many others (Additional file 2: Table S1).

Though MaMsvR only shares 33% identity with the previously described MthMsvR, they share a common DNA binding sequence motif. Additionally, the behavior of MaMsvR under non-reduced and reduced conditions represents a straightforward regulatory mechanism at its own promoter and represents a model for investigating the mechanism of MsvR family proteins and the role of the V4R domain cysteines in that mechanism.

### MaMsvR does not bind intergenic regions in a predicted *M. acetivorans* oxidative stress response operon

The *M. acetivorans* genes MA4664/MA3734-3743 comprise a putative operon encoding a variety of oxidative stress response proteins [28]. Although not apparent from the gene numbers, these genes are indeed adjacent on the chromosome (http://img.jgi.doe.gov) [28]. Since the MA3743 gene encodes a homologue of Mth FpaA, an F_420_H_2_ oxidase whose expression in *M. thermautotrophicus* is regulated by MthMsvR, we hypothesized that MaMsvR may regulate expression of this putative operon. However, EMSA did not show binding of MaMsvR to the upstream region of the 5′ gene in the putative operon (Figure 3c, Ma P_*4664*_, R). A second homologue of Mth FpaA is encoded by MA3381, which appears to be a monocistronic open reading frame. As with the putative oxidative stress operon, MaMsvR failed to bind the MA3381 upstream region in EMSA experiments (see Additional file 3: Figure S2a, b). These results implied that, unlike MthMsvR, MaMsvR might not be involved in regulating the expression of FpaA homologues. However, several other intergenic regions within the reported oxidative stress operon (MA4664/MA3734-3743) contain putative TATA box and BRE sequences that may represent alternate transcription start sites. To assess whether MaMsvR might be involved in regulating transcription from these sites, the upstream intergenic regions of the MA3734 and MA3736 genes were amplified and tested for MaMsvR binding by EMSA. The Ma histone A promoter (P_*hmaA*_) was used as a control to illustrate that MaMsvR binding is not non-specific. None of these regions exhibited any indication of MaMsvR binding (Figure 3c, P_*3734*_ and P_*3736*_, R lanes). Therefore, MaMsvR does not appear to directly regulate one of the putative oxidative stress operons in *M. acetivorans*.

Next, we tested whether MaMsvR might interact with any fragment of DNA containing the TTCGN_7-9_CGAA sequence that is important for MaMsvR binding to Ma P_*msvR*_. The Ma *rpoK* gene houses the MsvR binding motif within its open reading frame. MaMsvR did not bind to this template (Figure 3c, Ma *rpoK*, R lane), indicating that the presence of this sequence is not sufficient for MaMsvR binding. These results suggest that multiple factors, such as the surrounding promoter context [29], play a role in MaMsvR binding. Indeed, when the seventeen base pairs (<20% GC) on both sides of the MaMsvR binding sites are replaced with a different sequence (>40% GC) MaMsvR fails to bind (see Additional file 1: Figure S1). The additional flexibility in the DNA provided by the A-T rich sequence surrounding Boxes A and B may facilitate the binding of MaMsvR [30].

### Oligomeric state of MaMsvR

**Figure 4:**
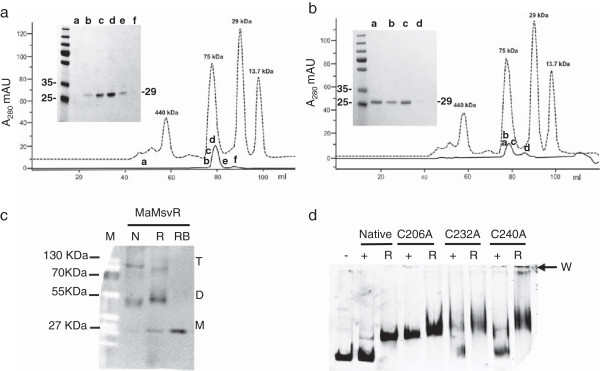
**Oligomeric Structure and the Role of Disulfide Bonds.** The dashed black line indicates the elution profile of the column calibration protein mix A (left to right: ferritin, conalbumin, carbonic anhydrase and ribonuclease A). The MaMsvR monomer is 29.2 kDa. (**a**) The elution profile for non-reduced MaMsvR (0.65 mg loaded) is indicated by the solid black chromatogram trace. Inset is an SDS-PAGE of MaMsvR fractions collected during the gel filtration run (a-f). (**b**) The elution profile for reduced (0.84 mg with 2 mM β-ME in the elution buffer) MaMsvR is indicated by the solid black chromatogram trace. Inset is an SDS-PAGE of MaMsvR fractions collected during the gel filtration run (a-d). (**c**) Immunoblot of an SDS –PAGE gel probed with a Strep-tag antibody where MaMsvR was prepared and subjected to electrophoresis (1 pmol each protein) in non-reducing SDS-PAGE sample buffer (N) and reducing (R) SDS-PAGE sample buffer on a 15% Tris-Glycine gel (no SDS). A reduced and boiled sample of MaMsvR is shown as a control (RB). The monomer is designated by M, whereas D and T indicate bands corresponding to a possible dimer and tetramer, respectively. (**d**) EMSA performed with Ma P_*msvR*_ and native MaMsvR and three C to A variants of MaMsvR. The control DNA only lane is indicated by a (-). The (+) lanes contain the indicated MaMsvR variant in the absence of any reducing agent. The (R) lanes contain the indicated MaMsvR variant and 5 mM DTT as a reducing agent.

Gel filtration chromatography was used to determine the oligomeric structure of non-reduced and reduced MaMsvR. MaMsvR^N-Strep^®^Tag^ was purified from *E. coli* under non-reducing or reducing conditions for these experiments. The molecular weight of the MaMsvR^N-Strep^®^Tag^ monomer is 29.2 kDa. Under non-reducing conditions, MaMsvR eluted from the gel filtration column with a size slightly larger than what was expected for a dimeric complex (Figure 4a, fractions b-e). SDS-PAGE analysis and staining of gel-filtration fractions confirmed the presence of MaMsvR (Figure 4a, inset). A small amount of UV absorbance was detected in the range for a monomer (Figure 4a, fraction f), but if this fraction did contain MaMsvR, the concentration was too low to be detected by SDS-PAGE (Figure 4a, inset). MaMsvR also eluted in the range of a dimeric complex under reducing conditions (2 mM β-ME) (Figure 4b) and SDS-PAGE confirmed the presence of MaMsvR in this peak (Figure 4b, inset). The peak had a longer tail than was present in the non-reducing samples, suggesting some MaMsvR monomer may have been present in the sample. However, only a faint band was detected by standard SDS-PAGE (Figure 4b and inset, fraction d). Taken together, these results suggest that MaMsvR predominantly exists as a dimer and that dimerization alone is not responsible for the differences in activity of non-reduced and reduced MaMsvR. Interestingly, the N-terminal region of MaMsvR contains a predicted dimerization interface that is characteristic of the ArsR family of transcription regulators and could facilitate dimerization ([19,31], Figure 1a, orange boxes).

The dimer may be further stabilized under non-reducing conditions by inter- or intra-chain disulfide bonds between cysteine residues of the C-terminal V4R domain. Such bonds have been proposed to form when transitioning from the non-reduced to the reduced state [9]. To test this possibility, MaMsvR was subjected to SDS-PAGE with and without DTT (in the absence of boiling), followed by Western blotting to visualize the different oligomers of MaMsvR (Figure 4c). A final concentration of 5 mM DTT was added to the reduced samples before electrophoresis; this is consistent with the concentration of DTT used in EMSA reactions. Without DTT and boiling, MaMsvR was primarily present as oligomers (Figure 4c, lane N). The smaller band (designated D) slightly below the 55 kDa marker was consistent with the predicted dimer size of 58.4 kDa [32]. The faint larger band suggested that a tetramer (designated by T) was formed in small amounts under non-reducing conditions (Figure 4c, lane N). The intensity of the band corresponding to a monomer (designated M) increased and the bands representing the dimer and tetramer were also present (Figure 4c, lane R) when DTT was added to the sample without boiling (Figure 4c, lane R). Since the SDS present in the sample-loading buffer should have disrupted the majority of non-covalent interactions even in the absence of boiling, disulfide bonds likely stabilized the observed oligomers.

Interestingly, under reducing conditions, the band in the dimeric range ran slower than the corresponding species under non-reducing conditions. Differences in the specific disulfide bonds formed under these conditions may have affected their compaction and altered their mobility through the gel. The large tetrameric complex also showed a slightly altered migration pattern under different conditions (Figure 4c, T). The tetrameric complex was not visible in gel filtration experiments under non-reducing or reducing conditions, perhaps due to a lower concentration of the oligomeric complex in the gel filtration samples compared to the sensitivity of protein detection in a western blot. It must be acknowledged that SDS-PAGE under the conditions utilized here is not immune to experimental artifacts, and the results must be interpreted with caution. Despite these limitations, the results observed with MaMsvR suggest disulfide bonds may be involved in conformational changes in the protein between the non-reduced form that does not bind Ma P_*msvR*_ DNA and the reduced form that does bind Ma P_*msvR*_ DNA. In anoxygenic phototrophic bacteria, oxidation results in the formation of disulfide bonds in the PpsR regulator, which leads to DNA binding and transcription repression [33].

### Role of V4R domain cysteines in MaMsvR function

Besides the three cysteines that are conserved in the V4R domain of MsvR family proteins, MaMsvR has seven additional cysteine residues (Figure 1a, gray boxes). With the exception of a cysteine at position 225, all non-conserved cysteines reside outside the V4R domain. Therefore, to further investigate the roles of the V4R domain cysteine residues (C206, C232, C240, Figure 1a, blue boxes, MaMsvR) in MaMsvR function, alanine substitutions of each cysteine were introduced using site-directed mutagenesis. EMSA analysis was performed with each of the MaMsvR^C→A^ variants to ascertain the impact of the substitution on MaMsvR binding to Ma P_*msvR*_ (Figure 4d). MaMsvR^Native^ only bound DNA under reducing conditions (Figure 2a; Figure 4d, left). MaMsvR variants had altered DNA binding profiles compared to the native protein, with MaMsvR^C206A^ having a clear impact on MaMsvR DNA binding. In contrast to MaMsvR^Native^, MaMsvR^C206A^ bound DNA under both non-reducing and reducing conditions (Figure 4d, C206A +, R lanes). The role of C232 and C240 in the transition from the non-reduced to reduced conformation was not as clear (Figure 4d). Both the MaMsvR^C232A^ and MaMsvR^C240A^ variants bound DNA under reduced conditions. However, the smearing of the bands indicated that the complexes were not stable [27,34]. Under non-reducing conditions, MaMsvR^C240A^ behaved more like the native protein whereas MaMsvR^C232A^ produced smearing and a shift similar to the reduced. The smearing for MaMsvR^C232A^ and MaMsvR^C240A^ was observed over multiple experiments suggesting that there is instability of the protein/DNA complex with these variants. When an alanine substitution was introduced at the fourth cysteine in the V4R domain, DNA binding did not differ from what was seen for the native protein indicating that this cysteine does not play a significant role in MaMsvR function (see Additional file 4: Figure S3).

The ability of C206A to bind DNA under non-reducing conditions suggests that the conversion from the non-Ma P_*msvR*_ DNA binding state (non-reduced) to the Ma P_*msvR*_ DNA binding state (reduced) involves at least one cysteine in the V4R domain. Furthermore, this data refuted the possibility that the lack of Ma P_*msvR*_ binding by MaMsvR^Native^ could be the result of non-specific disulfide bonds (involving any of the nine remaining cysteines) introduced during *in vitro* manipulations. However, the role of C232 and C240 in the transition from the non-reduced to reduced conformation is not as clear. C232 and C240 do appear to impact Ma P_*msvR*_ binding, but instability of the complexes suggests there may be other features of the protein that are impacted by the substitution.

### Mechanism of MaMsvR regulation at P_*msvR*_

**Figure 5:**
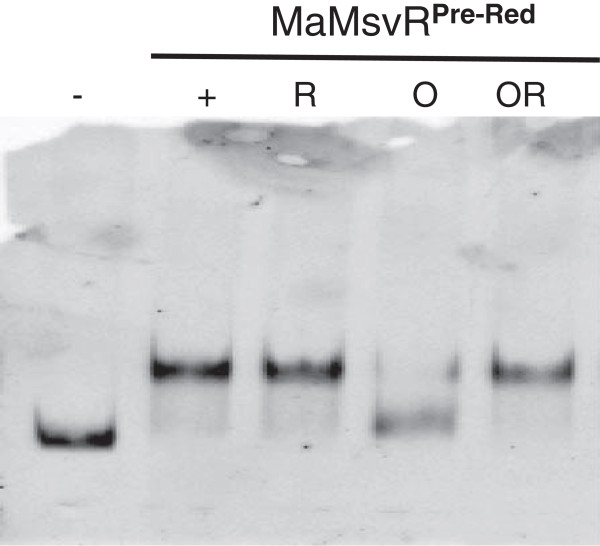
**Proposed Mechanism for Redox-Sensitive Transcriptional Regulation by MaMsvR.** EMSA experiment with pre-reduced MaMsvR and various treatments. The P_*msvR*_ DNA (10 nM) only control reaction is represented by (-). All other lanes contain P_*msvR*_ DNA (10 nM) and 200 nM MaMsvR^Pre-Red^ either in the absence (+, O) or presence (R, OR) of 5 mM DTT. Lanes labeled with (O) also contain 10 μM H_2_O_2_.

MaMsvR that has been pre-reduced (MaMsvR^Pre-Red^) [9] prior to use in EMSA assays bound to Ma P_*msvR*_ both in the absence or presence of DTT in the binding reaction. This binding is reversed by the addition of 10 μM H_2_O_2_ to a non-reduced (no DTT) binding reaction containing MaMsvR^Pre-Red^ (Figure 5, lane O). Subsequently, the addition of 5mM DTT to the H_2_O_2_ treated sample restored Ma P_*msvR*_ binding (Figure 5, lane OR). Together, the data presented herein suggest a mechanism by which MaMsvR may act as a redox-sensitive transcription repressor at its own promoter. In the reduced state, MaMsvR binds to and likely represses transcription from P_*msvR*_. Upon changes in redox conditions, MaMsvR undergoes a conformational change, rendering it unable to bind to the MsvR binding boxes [35]. Evidence presented herein suggest that the C206 residue of MaMsvR likely contributes to this conformational change.

## Conclusions

MaMsvR is a homologue of the previously characterized MthMsvR, and both proteins bind a characteristic TTCGN_7-9_CGAA motif that is present in the promoter regions of all MsvR homologues. In solution, MaMsvR is a dimer under non-reducing and reducing conditions. Both MaMsvR and MthMsvR exhibit differential DNA binding under non-reducing and reducing conditions. However, redox status has a far more obvious impact on MaMsvR, which binds DNA only under reducing conditions. Modification of cysteine residues in the V4R domain in an oxidizing environment likely results in conformational changes that interfere with MaMsvR binding to the Ma P_*msvR*_ DNA. Thus, derepression permits transcription under non-reducing conditions. There is an MsvR protein encoded in twenty-three of the forty fully sequenced genomes of methanogens, supporting an important, but poorly understood, role in methanogen biology. The results described here provide insight into the function and mechanism of MaMsvR, setting the stage for future investigation of MaMsvR regulated promoters using the *M. acetivorans* genetic system.

## Methods

### Reagents

T4 DNA ligase and Phusion™ DNA polymerase were purchased from New England Biolabs. Fast Digest ® restriction enzymes were purchased from Fermentas. General chemicals were purchased from Fisher Scientific.

### Sequence analysis

The *M. acetivorans* genome sequence (Accession number NC_003552) was downloaded into the Geneious software package [36]. All sequence manipulations were performed in Geneious and primers were designed using Primer 3 [37]. All DNA templates were confirmed by sequencing at the Oklahoma Medical Research Foundation.

### Transcription start site mapping

The transcription start site of Ma *msvR* was mapped using a 5′/3′ RACE kit (Roche Applied Science). All reactions were performed according to the manufacturers’ directions. Ma *msvR* specific cDNA was generated using 1 μg of total RNA and a gene specific primer (LK737, see Additional file 5). A control reaction lacking reverse transcriptase was performed to ensure any resulting amplification in later steps was not the result of contaminating chromosomal DNA. After A tailing the 3′ end of the cDNA with terminal deoxynucleotide transferase, a second gene specific primer (LK738, see Additional file 5: Table S2) was used to amplify the cDNA (in conjunction with a kit primer). The resulting amplicons were cloned into the pCR™-Blunt vector (Invitrogen) and sequenced using standard M13F and M13R primers.

### Cloning, expression, and purification of MsvR

The MaMsvR gene was PCR amplified with the primers LK588 and 589 (see Additional file 5: Table S2) containing a 5′ BamHI site and a 3′ PstI site, respectively, and cloned into an the pQE80L expression vector (Qiagen) modified with an N-terminal Strep-Tag®. The resulting plasmid was named pLK314 and transformed into *E.coli* Rosetta™ (Novagen) for expression. Cells were grown to an OD_600_ of 0.4 at 37˚C and then induced with 0.1 mM IPTG at 18˚C for 16 hours. Cells were lysed by sonication and the protein was purified with Streptactin resin (Qiagen) according to manufacturer’s recommendation. Reducing SDS-PAGE was employed to ensure no other proteins were present in MsvR preparations. Purified protein was dialyzed into a protein storage buffer (20 mM Tris pH 8, 10 mM MgCl_2_, 200 mM KCl, 25% glycerol) and stored at -20˚C. Protein concentrations were determined by the Bradford assay [38]. MaMsvR was diluted in the same protein storage buffer containing 50% glycerol to 2 μM for use in assays. MaMsvR was treated with 5 mM dithiothreitol (DTT) in reducing reactions. In non-reducing reactions, the protein samples were left untreated after aerobic purification. MthMsvR was purified and treated as previously described [9]. SDS-PAGE gels of representative purifications are shown in (see Additional file 6: Figure S4).

### MsvR V4R domain cysteine to alanine variants

Cysteine codons (TGT) were converted to alanine codons (GCT) using the QuikChange® site directed mutagenesis kit (Agilent Technologies). The sequence of primers used to generate individual alanine codon substitutions in pLK314 can be found in (see Additional file 5: Table S2). Plasmids resulting from QuikChange® reactions were confirmed by sequencing. The resulting MsvR variants were overexpressed and purified in the same manner as native MsvR.

### Electrophoretic mobility shift assay (EMSA)

Larger DNA templates for EMSA were PCR amplified from *M. acetivorans* C2A genomic DNA with custom primers (see Additional file 5: Table S2). With the exception of *rpoK* (MA0599) which is a portion of the open reading frame, all other templates (designated P_*xxxx*_) contain the extreme 5′ end of the predicted open reading frame and ~ 200 bp upstream of the translational start site. All templates were agarose gel purified, purified using the Wizard® SV PCR Clean-Up System (Promega), and confirmed by sequencing. DNA was quantified with the Quant-iT™ Broad Range DNA assay and a Qubit® fluorimeter (Invitrogen). Templates were diluted to 100 nM stocks for use in binding assays. The Mth templates were previously described [9,22]. Complementary oligonucleotides were annealed to generate the 50-bp DNA templates with mutations in the MsvR binding boxes (see Additional file 5: Table S2). Binding reactions and EMSAs were performed as previously described [9] with the exception that binding reactions were incubated at room temperature unless indicated otherwise. Gels were stained with SYBR® Gold Stain (Invitrogen) and visualized with a Gel Doc™ XR+ system (Bio-Rad). Image coloration was inverted for easier viewing.

### SDS-PAGE and western blotting

Protein samples were combined with an equal volume of 2X Laemmli sample buffer with or without a final DTT concentration of 5 mM and incubated at room temperature for five minutes. The protein samples were loaded with or without boiling on an AnykD™ gel (Bio-Rad) and electrophoresis was performed in 1X SDS-PAGE running buffer [39] alongside a PageRuler™ Prestained Protein Ladder Plus (Fermentas). After electrophoresis, proteins were transferred to Immun-Blot® PVDF membrane and transferred with a Mini Trans-Blot® cell (Bio-Rad) according to manufacturer recommendations. The membrane was probed with a Strep-tag antibody (Qiagen) and detected with the WesternDot™ 625 Western blot kit (Invitrogen). Membranes were visualized with a Gel Doc™ XR+ system (Bio-Rad).

### Size exclusion chromatography

Size exclusion chromatography was performed using a Superdex 200 HiLoad™ 16/600 column connected to an Äktapurifier UPC 10 (GE Healthcare). The running buffer consisted of 20 mM Tris pH 8, 10 mM MgCl_2_, 200 mM KCl and a 0.5 ml min^-1^ flow rate was used. The column was calibrated using a mixture of proteins from the low and high Molecular Weight GE Healthcare Gel Filtration Calibration kits. A protein mixture containing ferritin (440 kDa), conalbumin (75 kDa), carbonic anhydrase (29 kDa) and ribonuclease A (13.7 kDa) was prepared according to manufacturer instructions and used to calibrate the column (GE Healthcare). For molecular weight determination of non-reduced and reduced MaMsvR, 0.65 mg and 0.84 mg, respectively, were loaded onto the column in a volume less than 1 mL.

## Abbreviations

MsvR: Methanogen specific V4R domain regulator; SDS: Sodium dodecyl sulfate; EMSA: Electrophoretic gel mobility shift assay; PCR: Polymerase chain reaction; Mth: *Methanothermobacter thermautotrophicus*; Ma: *Methanosarcina acetivorans*; PAGE: Polyacrylamide gel electrophoresis; DTT: Dithiothreitol; β-ME: 2-mercaptoethanol.

## Competing interests

The authors declare that they have no competing interests.

## Authors’ contributions

CEI, JLT and EAK generated data in the laboratory. EAK and DJL were responsible for experimental design and manuscript preparation. All authors have read and approved of the final manuscript.

## Supplementary Material

Additional file 1: Figure S1 EMSAs with various mutations in Ma P_*msvR*_. Click here for file

Additional file 2: Table S1 Table of genes with potential MsvR binding sites upstream. Click here for file

Additional file 3: Figure S2 EMSAs with Ma P_*3381*_. Click here for file

Additional file 4: Figure S3 EMSA with MaMsvR^C225A^ Variant. Click here for file

Additional file 5: Table S2 Table of primers from this study. Click here for file

Additional file 6: Figure S4 SDS-PAGE of MsvR protein preparations. Click here for file
