# Supplementary material for: Redox-sensitive DNA binding by homodimeric Methanosarcina acetivorans MsvR is modulated by cysteine residues
Source: BMC Microbiol. 2013 Jul 16;13:163. doi: 10.1186/1471-2180-13-163 (PMC3729527; doi:10.1186/1471-2180-13-163)
Supplement: Additional file 1: Figure S1 — EMSAs with various mutations in Ma PmsvR. [file 1471-2180-13-163-S1.pdf]

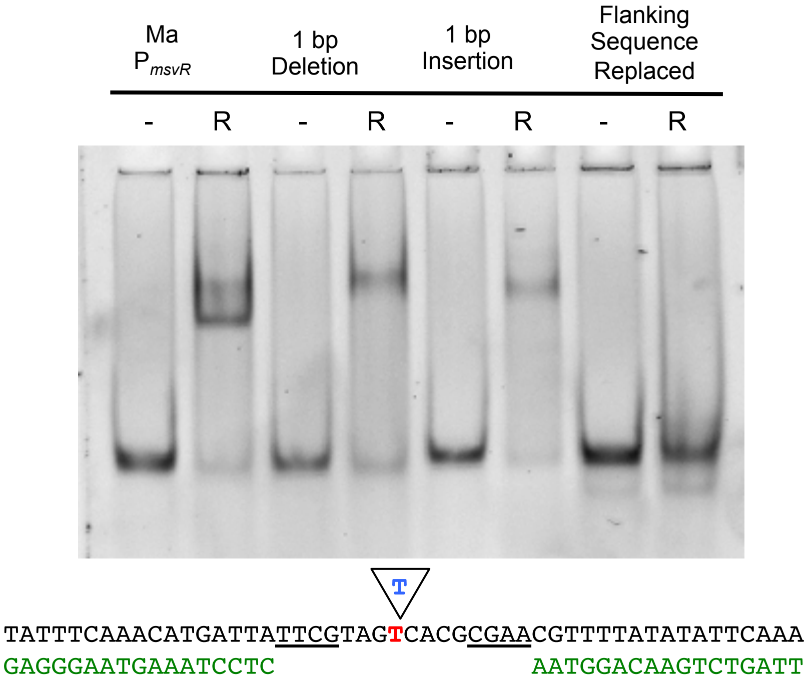

Figure S1. EMSAs with Ma P_{msvR} Mutations. MaMsvR was tested for binding to various mutant P_{msvR} templates under reducing conditions (R lanes, 5 mM DTT). DNA controls for each template are indicated by (-) labels above the gel. The deleted nucleotide in the spacer region is highlighted in red. The inserted nucleotide in the spacer region is in blue and the insertion site is indicated. The sequence of the exchanged flanking regions are indicated in green.
