# Supplementary material for: Redox-sensitive DNA binding by homodimeric Methanosarcina acetivorans MsvR is modulated by cysteine residues
Source: BMC Microbiol. 2013 Jul 16;13:163. doi: 10.1186/1471-2180-13-163 (PMC3729527; doi:10.1186/1471-2180-13-163)
Supplement: Additional file 3: Figure S2 — EMSAs with Ma P3381. [file 1471-2180-13-163-S3.pdf]

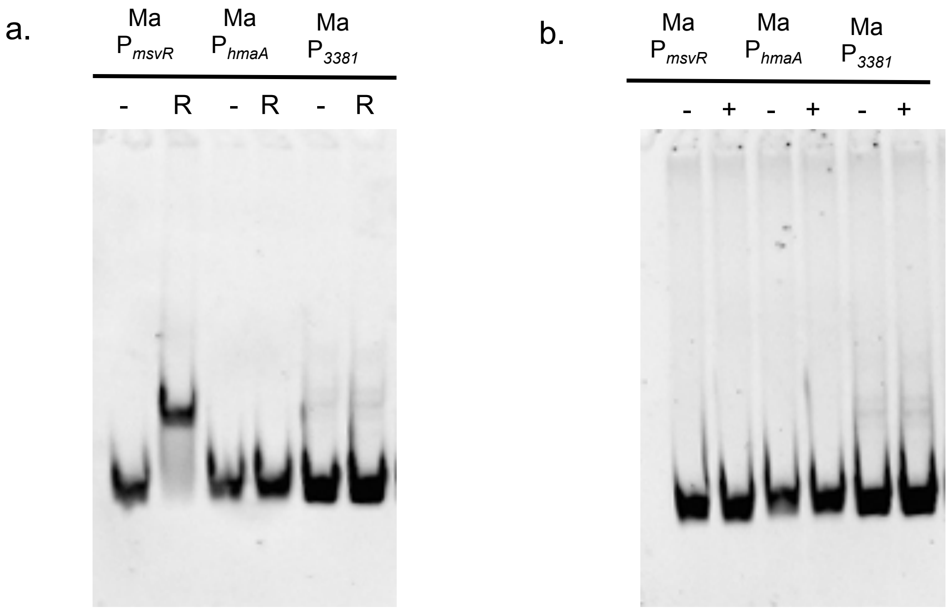


**Figure S2. EMSAs with Ma $P_{3381}$.** Ma $P_{3381}$ was tested for MaMsvR binding under reducing (a, R lanes) and non-reducing (b, + lanes). The (-) lanes are DNA only controls. Ma $P_{msvR}$ and $P_{hmaA}$ (histone A gene promoter) were used as positive and negative controls for MaMsvR binding.
