# Supplementary material for: Redox-sensitive DNA binding by homodimeric Methanosarcina acetivorans MsvR is modulated by cysteine residues
Source: BMC Microbiol. 2013 Jul 16;13:163. doi: 10.1186/1471-2180-13-163 (PMC3729527; doi:10.1186/1471-2180-13-163)
Supplement: Additional file 4: Figure S3 — EMSA with MaMsvRC225A Variant. [file 1471-2180-13-163-S4.pdf]

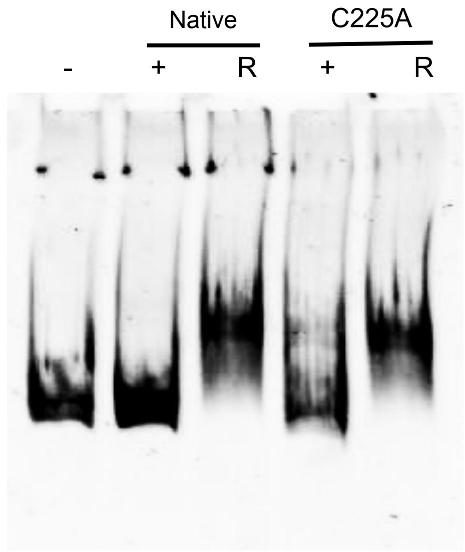

Figure S3. EMSA with MaMsvR^{C225A} Variant. EMSA with 10 nm Ma PmsvR and 200 nM native MaMsvR or 200 nM MaMsvR^{C225A}. The DNA only control lane is indicated by a (-). The (+) lanes contain DNA and protein and the (R) lanes contain DNA, protein, and 5 mM DTT.
