# Supplementary material for: Redox-sensitive DNA binding by homodimeric Methanosarcina acetivorans MsvR is modulated by cysteine residues
Source: BMC Microbiol. 2013 Jul 16;13:163. doi: 10.1186/1471-2180-13-163 (PMC3729527; doi:10.1186/1471-2180-13-163)
Supplement: Additional file 6: Figure S4 — SDS-PAGE of MsvR protein preparations. [file 1471-2180-13-163-S6.pdf]

a.

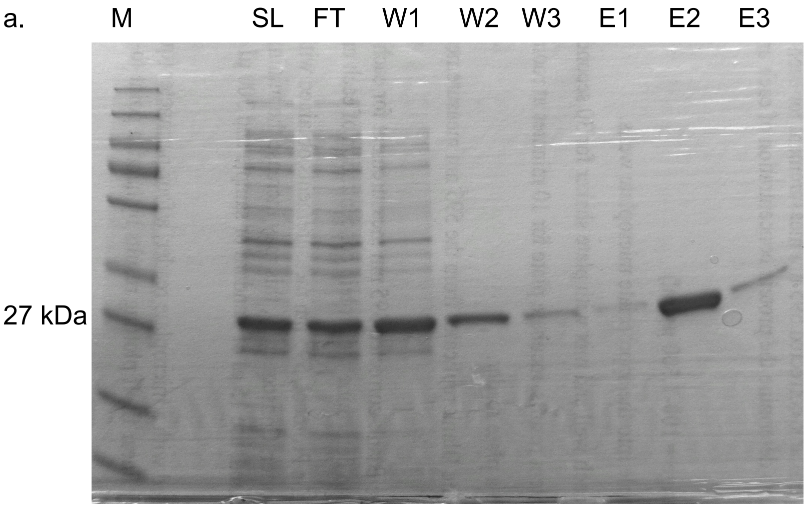

b.

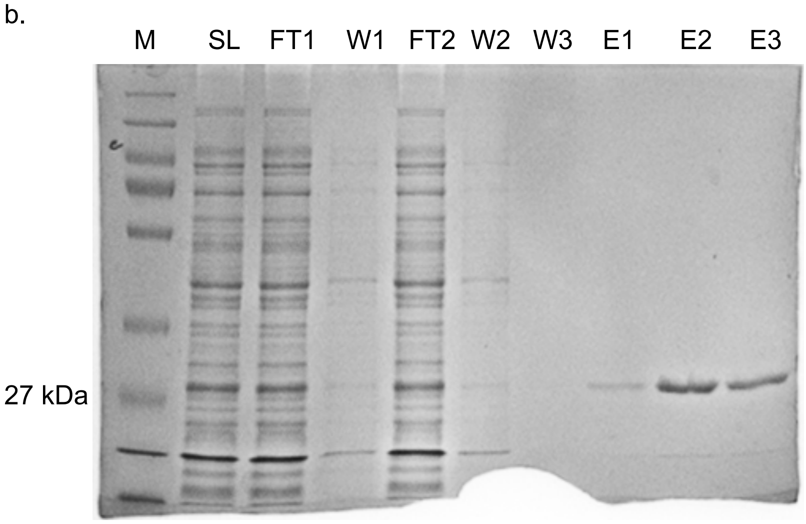

Figure S4. SDS-PAGE of MsvR Protein Purifications. Lane designations are marker, M; soluble lysate, SL; column flow through, FT; column washes, W; column elution, E. The 27 kDa marker band is indicated. Monomers of both MsvR homologues are 29-30 kDa. (a) Purification of MaMsvR. (b) Purification of MthMsvR.
